# Liver Function Tests Following Open Cardiac Surgery

**DOI:** 10.15171/jcvtr.2015.11

**Published:** 2015

**Authors:** Feridoun Sabzi, Reza Faraji

**Affiliations:** ^1^ Preventive Cardiovascular Research Center Kermanshah, Kermanshah University of Medical Sciences, Kermanshah, Iran; ^2^ Yazd Cardiovascular Research Center, Shahid Sadoughi University of Medical Sciences, Yazd, Iran

**Keywords:** Coronary Arteries Bypass Grafting, Livers, Hepatic Enzymes

## Abstract

***Introduction:*** The cardiopulmonary bypass may have multiple systemic effects on the body organs as liver. This prospective study was planned to explore further the incidence and significance of this change.

***Methods:*** Two hundred patients with coronary artery bypass grafting (CABG), were randomly selected for the study. Total and indirect bilirubin, aspartate aminotransferase, alanine aminotransferase, alkaline phosphatase were measured preoperatively and at 24, 48 and 72 hours, following coronary artery bypass grafting. Postoperative value of the liver function tests with respect to hypothermia or hypotension were compared by one way analysis of variance for repeated measure and compared with t test. Patient’s characteristics with bilirubin value (≤1.5 mg or >1.5 mg) were compared with t test.

***Results:*** A significant increase of total bilirubin, aspartate aminotransferase, and alkaline phosphatase were noted in the third postoperative day. Significant relation was seen between hypotension and alkaline phosphatase, and aspartate aminotransferase change but hypothermia had not affected alanine aminotransferase, total bilirubin and indirect bilirubin change. Pump time, alanine aminotransferase in third postoperative day and direct bilirubin in first and second day of postoperative period had significant relation with pre and post-operative bilirubin change.

***Conclusion:*** Transient but not permanent alterations of hepatic enzymes after coronary artery bypass grafting presumably attributed to the decreased hepatic flow, hypoxia, or pump-induced inflammation.

## Introduction

The institution of cardiopulmonary bypass (CPB) in open cardiac surgery is associated with some body organ dysfunction.^1^ As the number of patients undergoing cardiac surgery is increased need to CPB is also increased. CPB is non physiologic circulation and the patients are subjected to a various degree of body organ dysfunction.^1,2^ The careful recent medical literature review revealed that most studies have been focused on methods to protect organs from adverse effect of CPB; however, most of these studies missed evaluation of the liver function and focused on organs such as heart, lung and kidney. Paparella et al^3^ showed that in the high-risk cardiac surgery, about 10% of patients who received CPB, experience hepatic injury, which directly affects morbidity and mortality of patients. Wark supposed two main pathophysiologic mechanisms for the post CPB hepatic injury that includes the systemic inflammatory response syndrome and oxidative stress.^4^ However, Edmunds exhibits that most of these pathophysiologic mechanisms are based on centrilolular sinusoid ischemia and subsequent reperfusion injuries. Others mechanism that propounded by Hayashida et al^5^ include drug induced hepatic injury or systemic inflammatory reaction by CPB. High mortality of post CPB liver injury needs to convenience mechanisms of hepatic injury induced by CPB. Some trials have shown that the consumption of coagulation factors during CPB leads to reduced coagulation factors and compromised liver function by micro thrombi formation in centrilolular hepatic sinusoids.^6^ On the other hand, in some controversial studies, short CPB time less than two hours were not associated with compromised hepatic function test.^7^ Furthermore, an elevation of serum liver enzymes after uncomplicated CPB has been reported in some studies. These changes could to be attributed to splanchnic ischemia caused by centrilobular hepatic cell ischemia and shocked liver.^8^ This study was planned to explore, the incidence and clinical significance of serum liver test changes after CPB.

## Patients and Methods

A prospective study was carried out at our center, 400 patients scheduled to undergo first time coronary artery bypass grafting (CABG) who had full criteria in entering to study, were randomized, with a computer generated random number, patients were consecutively allocated to singular or plural nouns. One hundred patients with singular number and 100 patients with plural number entered to study. The exclusion criteria were set up in order to eliminate any other known cause of possible liver function disturbances that includes right heart failure, combination of valve and coronary artery disease (CAD), Gilbert syndrome, acute inflammation of gallbladder or any other complication of gallstone disease, active viral and non viral hepatitis and, hematological disorders, toxic anesthesia drugs and incomplete data. The operative technique was similar in all patients. General anesthesia was induced and maintained with total intravenous anesthesia, consisting of 2% propofol (100 µg/kg/min) and remifentanil (0.15 µg/kg/min). And atracurium (5 µg/kg/min) was infused intravenously. After anesthesia, a median sternotomy was performed, followed by routine aortic and atrial cannulation. CPB was established using membrane oxygenation, non-pulsatile perfusion, and moderate systemic hypothermia. Myocardial protection was achieved by cold hyperkalemic crystalloid cardioplegia and topical cooling with iced slush and cold saline solution. Cardioplegia was administered in a retrograde and antegrade fashion in all patients. CABG was performed using the left internal mammary artery and reversed saphenous vein in most instances. Transfusion of packed red blood cell concentrates was accomplished when the hematocrit or hemoglobin (Hct/Hb) value was less than 0.20/7 g/dl. In the open heart surgery intensive care unit (ICU), blood transfusion was performed when a Hct/Hb level reach to 0.25/8 g/dl or less. Postoperative transfusion of fresh frozen plasma, platelets or cryoprecipitate was fulfilled in the presence of active bleeding (more than 200 ml/h in 3 consecutive hours) and presence of coagulation disturbances (platelet count less than 80 × 10^9^, PT or PTT >1.5 × of reference laboratory value or fibrinogen level less than 1 g/l. Cell saver machine and auto transfusion system were not applied in ICU. Packed red blood cells were used during CPB time as the patient’s hemoglobin reduced to 7.0 g/dl. Postoperative indication for packed red blood cells includes: (1) hemoglobin <8.0 g/dl, (2)excessive mediastinal bleeding more than 600 ml in three consecutive hours. Others blood products, such as platelets and fresh frozen plasma and cryoprecipitate were transfused as patient’s condition warranted. Five liver function parameters were measured preoperatively and at 24, 48 and 72 hours following surgery: Total and indirect bilirubin (TB-IB), aspartate aminotransferase (AST), alanine aminotransferase (ALT), alkaline phosphatase (ALP). Results were expressed as mean value ± standard deviation. Biochemical analysis (TB, IB, and liver enzymes) was performed using an automated analyser (Biolis 24 i premium, Tokyo Boeki, medical system). We decided to distinguish patients with bilirubin of ≤1.5 mg from those with bilirubin of more than 1.5 mg with respect to preoperative, intraoperative and postoperative variables. Strict exclusion criteria were adopted in order to eliminate any known cause of possible liver function disturbances. The postoperative value of liver function test (LFT) in three consecutive postoperative days with respect to hypothermia or hypotension was compared by one way analysis of variance (ANOVA) for repeated measures and the preoperative value of liver function test compared with postoperative (three consecutive days) with *t* test. Patients’ characteristics were compared using *t* test for continuous variables (preoperative liver enzymes, TB, IB, operative time and transfusion volume). In the subgroups of the patients with or without hyperbilirubinemia, *t* test and chi-square test were used for continuous and categorical variables on each post operative day, consequently. Statistical analysis was performed with the statistical package for the social sciences (SPSS 11.5). *P*-values less than 0.05 were considered statistically significant.

## Results

Totally, two hundred patients fulfilled the criteria and randomly were included in the study. There were 120 women (60%) and 80 men (40%), with a mean age of 58 ± 5 years. The mean duration of the CPB time was 66 ± 8 minutes. The mean duration of the aortic cross clamp time was 44±5 minutes. The preoperative TB (1 ± 0.4 mg/ml) was increased up to 20% in third postoperative day (1.2 ± 0.4 mg/ml) but there was not significant differences between preoperative value and first and second postoperative day. Likewise, the preoperative IB (0.35 ± 0.2 mg/ml) was decreased up to 45% in third postoperative day (0.22 ± 0.2 mg/ml). This difference was not statistically significant between preoperative value and first and second day. The changes in the level of liver amino transferase enzymes were more remarkable. The preoperative AST (18 ± 11 U/ml) was increased up to 7.3% in third postoperative day (25 ± 12 U/ml; *P *= 0.001). The preoperative ALT (47 ± 14 U/ml) was increased up to 4% in third postoperative day (52 ± 10 U/ml; *P* > 0.05). The preoperative alkaline phosphatase (120 ± 61 U/l) was increased up to 34% in third postoperative day (178 ± 88 U/l; *P *= 0.01; Table 1 and 2), but was not changed in first and second post operative day. In one way analysis of variance, effect of hypotension on quantity of postoperative ALT, AST, ALP, TB and IB was significant in first, third, second, first, second postoperative day, consequently (*P *< 0.05; Table 3 and 4). There was significant correlation between hypothermia and quantity of AST, ALT and ALP in the first postoperative day but could not be attributed to the degree of direct and indirect bilirubin in first postoperative day. There was significant relation between prolonged pump time (>100 minutes) and quantity of AST in the second postoperative day but prolonged pump time has not effect on others postoperative liver function test. Correlation between transfusion (>6 unite) and quantity of ALP and ALT were significant in first postoperative day but this correlation with TB was significant in the second postoperative day. Prolonged aortic cross clamp time has not any effect on postoperative LFT.

**Table 1:** Comparison of Pre and Postoperative Liver Function Test Value by *T* Test

**Value**	**Total Bilirubin**	**Indirect Bilirubin**	**Aspartat Aminoteransferase**	**Alanine Aminotransferase**	**Alkaline Phosphatase**
Preoperative value (mean±SD)	1±0.4	0.35±0.2	18±11	47±14	120±61
First postoperative day	1±0.5	0.34±0.32	20±8	52±28	127±77
Second postoperative day	1 ±0.7	0.3±0.2	20±10	47±23	123±92
Third postoperative day	1.2±0.4^a^	0. 2±0.2	22±15^a^	52±10	178±88^a^
*P*-value	<0.05	<0.05	<0.05	>0.05	<0.05

^a^Significant.

**Table 2:** Effect of Hypotension on Postoperative Liver Function Test by Two Way ANOVA

**Hypotension**	**Total Billirubin**	**Indirect Billirubin**	**Aspartat Aminoteransfrase**	**Alanine Aminotransferase**	**Alkaline Phosphatase**
First postoperative day	1±0.5	0.4±0.6	20±8	52±28	127±77
Second postoperative day	1.2±0.7	0.3±0.2	20±10	47±23	123±92
Third postoperative day	2.1±0.9	0. 2±0.2	22±15^a^	22±10	178±88^a^

^a^Significant.

**Table 3:** Effect of Hypothermia on Postoperative Liver Function Test by One Way ANOVA

**Hypothermia**	**Total Billirubin**	**Indirect Billirubin**	**Aspartat Aminoteransfrase**	**Alanine Aminotransferase**	**Alkaline Phosphatase**
First postoperative day	1±0.5	0.4±0.6	20±8	52±28	127±77
Second postoperative day	1.2±0.7	0.3±0.2	20±10	47±23	123±92
Third postoperative day	2.1±0.9^a^	0. 2±0.2	22±15	22±10	178±88^a^

^a^Significant.

**Table 4:** Comparison of Patient’s Characteristics Between Two Groups

**Variable**	**Bilirubin ≤1.5**	**Bilirubin >1.5**	*** P *** **-value**
Age(Mean± SD)	52±11	52±14	>0.05
Volume of transfused blood	3.2±1.2	3.5±0.9	>0.05
Hypothermia	28±1.4	29±1.2	>0.05
Pump time	94±25	107±36	0.05
Preoperative SGOT	46.9±34	51±26	>0.05
SGOT^a^	50±26	61±33	>0.05
SGOT^b^	45±23	52±22	>0.05
SGOT^c^	21±7	28±17	<0.05
Preoperative SGPT	18±10	20±15	>0.05
SGPT^a^	19±8	22±7	>0.05
SGPT^b^	19±9	24±15	>0.05
SGPT^c^	21±13	27±21	>0.05
Preoperative Alkaline phosphatase	123±67	109±31	>0.05
Alkaline phosphatase^a^	130±87	18±21	>0.05
Alkaline phosphatase^b^	127±102	108±33	>0.05
Alkaline phosphatase^c^	181±98	169±43	>0.05
Preoperative Total bilirubin	0.9±0.28	2±0.5	<0.05
Total bilirubin^a^	1.07±0.5	1.9±0.8	<0.05
Total bilirubin^b^	0.9±0.3	1.6±0.65	<0.05
Total bilirubin^c^	2.4±1.5	1.3±0.5	>0.05
Preoperative direct bilirubin	0.2±0.5	0.7±0.1	<0.05
Direct bilirubin^a^	0.3±0.6	0.6±0.5	<0.05
Direct bilirubin^b^	0.2±0.1	0.5±0.4	>0.05
Direct bilirubin^c^	0.17±0.2	0.2±0.2	>0.05
Gender (male)	67.5%	77.3%	>0.05
Gender (female)	32.5%	27.7%	>0.05
Intraoperative hypotension	29.9%	27.3%	>0.05
Anesthesia time	7.8±2	7.3±2	>0.05

^a^First post operative day. ^b^ Second post operative day. ^c^Third post operative day.

## Discussion

The pathogenesis of liver dysfunction after cardiac surgery is multi-factorial. Varghese et al^6^ stated that alterations in hepatic blood flow to be a major factor in pathogenesis of postoperative hepatic function dysfunction. It has also been proposed that factors related to CPB such as activation of inflammatory system pose an additional risk for hepatic dysfunction. In our study, pump time has significant effect on hepatic function tests via hypothermia or hypotension (Table 3). Braun et al^7^ concluded that increased oxygen extraction during CPB may indicate an inflammatory reaction due to the CPB that beginning in the vascular livers bed and others abdominal organ and redistribution of the splanchnic blood flow during the CPB. Normothermic CPB does not lead to a significant or prolonged reduction of liver function but causes an increase of gastrointestinal permeability. The increased gastrointestinal permeability caused sepsis and multi organ failure.^8^ Hypothermic CPB may benefit the hepatic circulation, although the additional advantages usually gained by the use of pulsatile perfusion may be partly lost when hypothermia is combined with a high pump flow rate. In our study, in ANOVA, hypothermia significantly affected total bilirubin and alkaline phosphates levels on third post operative day. In contrast to others study ^6-9^ advantage of hypothermia may be lost when the effect of hypothermia was considered combined with others variables likes hypotension and prolonged operation time (Table 4). Laupacis et al^10^ and Robinson et al^11^ proposed that hepatic sinusoidal endothelial cells are more vulnerable to hypoxia or hypothermia than hepatocytes, and concluded that hepatosplanchnic oxygen consumption and extraction increased during normothermia. In our study effect of hypothermia on hepatic enzymes were evaluated in three successive days. The hypothermia (less than 28), has significant influence on ALT in the third postoperative day, however, normothermia has significant effect on AST on second post operative day. Results of our study showed that most sensitive test for postoperative evaluation of hepatic function in hypothermic and normothermic CPB is AST and ALT consequently. In Wang study,predictors of possible risk factors for postoperativehyperbilirubinemia were the numbers of replaced valves,preoperative right atrial pressure, and preoperative TBconcentration.^12^ We believe that high right atrial pressure in Wang study caused hepatic congestion and centrilolular cell necrosis and jaundice. Ninety percent of theincrease in serum bilirubin was due to a rise in unconjugated bilirubin on the first postoperative day. Preoperative TB concentration and preoperative high right atrial pressure were the factorsassociated with increased risk of postoperative hyper bilirubinemiaby logistic regression analysis. The incidence of postoperative jaundice in our study was much lower than others study. The high incidence of postoperative jaundice in Abha study related to prolonged pump time in comparison to shorter pump time in our study.^13^ In Collins et al^14^ study hypotension, hypoxia, hemolysis, heart failure and hypothermia were not associated with the development of post operative jaundice, although post operative jaundice was significantly associated with multiple valve replacement, higher transfusion requirements, and longer CPB. We believe that excretion of bilirubin related to hepatocyte normal function, and, occurrences of centrilolular cell necrosis exacerbates postoperative hepatocyte dysfunction Jakob et al^15^ hypothesized that increasing pump flow during hypothermic CPB would improve organ perfusion and reduce the inflammatory response in the post-operative period. In our study, type of CPB has not any influence on the post operative LFT. This is one of the important limitations of our study, because we have not included complex and combined surgery in our study, and short pump time and cross clamp time would not reveals, effect of specific pump type on blood cell components. Strict exclusion criteria have been set in our study in order to exclude patients with known liver function test abnormality, or any other condition, which could affect hepatic enzymes postoperatively such as chronic obstructive pulmonary disease COPD. The patients with severe COPD have some degree of right ventricular (RV) failure. RV failure caused hepatic congestion and exacerbates effect of CPB and intraoperative hypotension on liver function test. This conflict was not considered in inclusion criteria in previously mentioned studies.^16^ Our results showed a statistically significant increase of total bilirubin and decrease of indirect bilirubin. Transferees enzymes were also significantly increased (AST, ALP). Dancona et al.^17^ have shown that in some patients, CPB caused hypo perfusion. Effects of hypo perfusion and hypoxia on intestinal cells lead to activation of hepatic macrophages. Consecutive releases of mediators by macrophages lead to the development of jaundice and multiple organ failure. Vazquez et al^18^ demonstrated predicting factors in shocked liver and right heart failure following CPB include: hypotension, hypoxia, hypothermia and liver congestion. In these circumstances liver cannot handle the bilirubin load presented by massive transfusions. Transient postoperative high hepatic enzymes levels also may be seen in massive transfusion associated with low cardiac output. Prolonged low cardiac output, which may compromise the hepatic blood flow, caused alterations in the microcirculation.^19-21^ We believe that there are two type of CPB induced hepatic failure. In type І, centrilolular and sinusoidal cell necrosis caused hypotension or shocked liver syndrome. In this condition, the patients may or not have acute renal failure but definitely have not end organ vasculitis. In type 2, there are generalized end organ vasculitis such as acral part vasculitis, that caused fingers and toes hypo perfusion and renal, brain or hepatic micro vascular involvement and leads to acute renal failure, encephalopathy and liver failure. The second type of CPB-induced vasculitis is very rare and in our knowledge careful literature review revealed few documented cases. Ohri and Velissaris^22^ demonstrated that administration of inotropic drugs further depresses portal vein flow while hepatic artery flow increases slightly. The net effect is a further decrease in total hepatic blood flow. Hessel^23^ also proposed that CPB induced hypotension or postoperative low cardiac output causes splanchnic venous spasm. Sanderson et al^24^ demonstrated that during CPB, total blood flow has been shown to decrease by approximately 20% and hepatic arterial blood flow by 20% to 45%. Olsson et al^25^ and Collins et al^14^ stated that CPB-induced hepatic dysfunction may interfere withliver scavenger function for gut derived bacteria and cytokines, leading to endotoxinemia andsepticemia an important risk factor for systemic inflammatoryresponse syndrome and subsequent multiple organ failure. The incidence of postoperativehyperbilirubinemia in Faust and Reddy ^26^ study was 35.1%, opposed to 7% in our study. Despite progress in technology of oxygenators, techniques of surgery,CPB, and cardiac anesthesia in the last decades variability in incidence of post operative jaundice related to surgeon experiences likes short pump time.^27,28^ In our study, mortality of thepatients with CPB induced jaundice was 15%, opposed to only 5.6% in other study.^27^ In the Collins et al^14^ study the serum totalbilirubin concentration, mainly resulting from increased conjugatedbilirubin, which reached a maximum level on the second postoperativeday. Collins et al^14^ supposed that this single serum total bilirubin measurementon the second postoperative day could identify patients at highrisk for mortality in the postoperative period. In our study, the serum total bilirubin reached its maximum level on the first, second and third postoperative day in 40%, 50%, and 10% of the patients successively, and increased serumtotal bilirubin came mainly from unconjugated bilirubin. Thecorrelation of postoperative hyperbilirubinemia with higherpostoperative mortality was also noted in our study. The mortality of patients are related to the time of postoperative jaundice occurence^28^ and was significantly increased in patients whose total bilirubin concentrationreached to its peak level on the seventh postoperative day.The mortality is lowerin the patients whose total bilirubin concentration increased topeak level on the first and second post operative day. In our study the mortality was higher when peak postoperative jaundice reached to peak level on the third postoperative day. Nature of the hyperbilirubinemia, would determine the course and mortality of CPB-induced jaundice. Collins et al^14^ supposed that failure of thecanalicular excretion of bilirubin was the main causeof this conjugated hyperbilirubinemia. In contrast to previous study, postoperative hyperbilirubinemiacomes mainly from an increase of unconjugated bilirubin withhemolytic origin in 50 patients who underwent cardiac operations in our study.In Klepetko and Miholic study,^29^ 70% of the increase intotal bilirubin after operation comes primarily from unconjugated bilirubin. Chu et al^30^ stated that significant decrease of haptoglobin concentrationwith lactate dehydrogenises increasing immediately after operations suggests thatpostoperative hyperbilirubinemia is caused by an unconjugatedbilirubin load. Mathie^27^ stated that mechanisms underlying the development of late postoperativehyperbilirubinemia differ from those of the earlypeak total bilirubin level. They supposed that immediate occurrence and rapid decline of postoperativehyperbilirubinemia related both to hepatic canalicular failure for disposing bilirubin and in other hand, may reflectthe transient damaging effects on the blood elements whereas steady increase of the total bilirubin levelto reach its peak on the seventh day, mainly conjugated bilirubin,was a consequence of hepatic dysfunction caused by factors such as Centrilobular cell necrosis, hepatic congestion, right or bi ventricular cardiac failure.^31,32^

## Acknowledgments

We extend our sincere appreciation to the efforts taken by thestaff of the Department of Cardiovascular Surgeryin Imam Ali hospital that without whose efforts we would not have been able to completethis project.

## Ethical Issues

The study was approved by the Local Ethics Committee.

## Competing Interests

Authors declare no conflict of interest in this study.

## References

[1] Kawahira T, Wakita N, Minami H (2003). Lymphatic cardiac tamponade after open-heart surgery. Jpn J Thorac Cardiovasc Surg.

[2] Welbourn N, Melrose DG, Moss DW (1966). Changes in serum enzyme levels accompanying cardiac surgery with extracorporeal circulation. J Clin Pathol.

[3] Paparella D, Yau TM, Young E (2002). Cardiopulmonary bypass induced inflammation: pathophysiology and treatment An update. Eur J Cardiothorac Surg.

[4] Wark HJ (2002). Hepatic failure after cardiopulmonary bypass is unlikely to be isoflurane hepatitis. Anesthesiology.

[5] Hayashida N, Shoujima T, Teshima H (2004). Clinical outcome after cardiac operations in patients with cirrhosis. Ann Thorac Surg.

[6] Varghese D, Varghese B, Kelley K (2007). Prospective randomized study to evaluate changes in alpha-GST as a novel marker of hepatocellular necrosis in patients at high-risk of hepatic injury undergoing coronary revascularization with and without cardiopulmonary bypass. Ind J Thorac Cardiovasc Surg.

[7] Braun JP, Schroeder T, Buehner S (2004). Splanchnic oxygen transport, hepatic function and gastrointestinal barrier after normothermic cardiopulmonary bypass. Acta Anaesthesiol Scan.

[8] Autschbach R, Falk V, Lange H (1996). Assessment of metabolic liver function and hepatic blood flow during cardiopulmonary bypass. Thorac Cardiovasc Surg.

[9] Ascione R, Talpahewa S, Rajakaruna C (2006). Splanchnic organ injury during coronary surgery with or without cardiopulmonary bypass: A randomized, controlled trial. Ann Thorac Surg.

[10] Laupacis A, Fergusson D (1997). Drugs to minimize perioperative blood loss in cardiac surgery: metaanalysis using perioperative blood transfusion as the outcome. Anesth Analg.

[11] Robinson RM, Holland P, Schmidt PJ (1965). Serum Hepatitis After Open-Heart Surgery. J Thorac Cardiovasc Surg.

[12] Wang MJ, Chao A, Huang  CH (1994). Hyperbilirubinemia after cardiac operation Incidence, risk factors, and clinical significance. J Thorac Cardiovasc Surg.

[13] Abha C, Debasish G, Sri S (1999). Hyperbilirubinemia After Cardiopulmonary Bypass. Asian Cardiovasc Thorac Ann.

[14] Collins JD, Bassendine MF, Ferner R (1983). Incidence and prognostic importance of jaundice after cardiopulmonary bypass surgery. Lancet.

[15] Jakob SM, Ruokonen E, Takala J (2000). Assessment of the adequacy of systemic and regional perfusion after cardiac surgery. Br J Anaesth.

[16] Cohen JA, Kaplan MM (1978). Left-sided heart failure presenting as hepatitis. Gastroenterology.

[17] Dancona G, Baillot R, Poirier B (2003). Determinants of gastrointestinal complications in cardiac surgery. Tex Heart Inst J.

[18] Vazquez P, Lopez J, Carrillo A (2001). Hepatic dysfunction after cardiac surgery in children. Pediatr Crit Care Med.

[19] Bohmer T, Kjekshus E, Nitter S (1994). Studies on the elimination of bilirubin pre-operatively in patients with mitral valve disease. Eur Heart J.

[20] Desai JB, Ohri SK (1990). Gastrointestinal damage following cardiopulmonary bypass. Perfusion.

[21] Bynum TE, Boitnott JK, Maddrey WC (1979). Ischemic hepatitis. Dig Dis Sci.

[22] Ohri SK, Velissaris T (2006). Gastrointestinal dysfunction following cardiac surgery. Perfusion.

[23] Hessel EA (2004). Abdominal Organ Injury After Cardiac Surgery Seminars in Cardiothoracic and Vascular Anesthesia,. Asian Cardiovasc Thorac Ann.

[24] Sanderson RG, Ellison JH, Benson JA (1967). Jaundice following open heart surgery. Ann Surg.

[25] Olsson R, Hermodsson S, Roberts D (1984). Hepatic dysfunction after open-heart surgery. Scand J Thorac Cardiovasc Surg.

[26] Faust TW, Reddy KR (2004). Postoperative jaundice. Clin Liver Dis.

[27] Mathie RT (1993). Hepatic blood flow during cardiopulmonary bypass. Crit Care Med.

[28] Mukamal KJ, Girota S, Mittleman MA (2006). Alcohol consumption, atherosclerotic progression, and prognosis among patients with coronary artery bypass grafts. Am Heart J.

[29] Klepetko W, Miholic J (1985). Jaundice after open heart surgery: a prospective study. Thorax.

[30] Chu CM, Chang CH, Liaw YF (1984). Jaundice after open heart surgery: a prospective study. Thorax.

[31] Kumle B, Boldt J, Suttner SW (2003). Influence of prolonged cardiopulmonary bypass times on splanchnic perfusion and markers of splanchnic organ function. Ann Thorac Surg.

[32] Klemperer JD, Ko W, Krieger KH (1998). Cardiac operations in patients with cirrhosis. Ann Thorac Surg.

